# Up-regulation on cytochromes P450 in rat mediated by total alkaloid extract from *Corydalis yanhusuo*

**DOI:** 10.1186/1472-6882-14-306

**Published:** 2014-08-18

**Authors:** Jingjing Yan, Xin He, Shan Feng, Yiran Zhai, Yetao Ma, Sheng Liang, Chunhuan Jin

**Affiliations:** School of Chinese Materia Medica, Tianjin University of Traditional Chinese Medicine, 312 Anshanxi Road, 300193 Tianjin, Nankai District, China; Tianjin State Key Laboratory of Modern Chinese Medicine, 312 Anshanxi Road, 300193 Tianjin, Nankai District, China

**Keywords:** Total alkaloid extract, Cytochromes P450, Histopathology, Enzyme activity, mRNA level, Herb (drug)-drug interaction

## Abstract

**Background:**

Yanhusuo (*Corydalis yanhusuo* W.T. Wang; YHS), is a well-known traditional Chinese herbal medicine, has been used in China for treating pain including chest pain, epigastric pain, and dysmenorrhea. Its alkaloid ingredients including tetrahydropalmatine are reported to inhibit cytochromes P450 (CYPs) activity in vitro. The present study is aimed to assess the potential of total alkaloid extract (TAE) from YHS to effect the activity and mRNA levels of five cytochromes P450 (CYPs) in rat.

**Methods:**

Rats were administered TAE from YHS (0, 6, 30, and 150 mg/kg, daily) for 14 days, alanine aminotransferase (ALT) levels in serum were assayed, and hematoxylin and eosin-stained sections of the liver were prepared for light microscopy. The effects of TAE on five CYPs activity and mRNA levels were quantitated by cocktail probe drugs using a rapid chromatography/tandem mass spectrometry (LC-MS/MS) method and reverse transcription-polymerase chain reaction (RT-PCR), respectively.

**Results:**

In general, serum ALT levels showed no significant changes, and the histopathology appeared largely normal compared with that in the control rats. At 30 and 150 mg/kg TAE dosages, an increase in liver CYP2E1 and CYP3A1 enzyme activity were observed. Moreover, the mRNA levels of CYP2E1 and CYP3A1 in the rat liver, lung, and intestine were significantly up-regulated with TAE from 6 and 30 mg/kg, respectively. Furthermore, treatment with TAE (150 mg/kg) enhanced the activities and the mRNA levels of CYP1A2 and CYP2C11 in rats. However, the activity or mRNA level of CYP2D1 remained unchanged.

**Conclusions:**

These results suggest that TAE-induced CYPs activity in the rat liver results from the elevated mRNA levels of CYPs. Co-administration of prescriptions containing YHS should consider a potential herb (drug)–drug interaction mediated by the induction of CYP2E1 and CYP3A1 enzymes.

## Background

*Corydalis yanhusuo* W.T. Wang, belonging to the *Papaveraceae* family, is a well-known traditional Chinese herbal medicine. The dried and pulverized tubers of *C. yanhusuo* are called Yanhusuo (YHS). It is among the 50 fundamental herbs in Chinese herbology and has been used traditionally for its sedative, neuroleptic, and analgesic properties [1]. Alkaloids are the main active constituents isolated from YHS [2], and studies have recently shown that these have extensive pharmacological activities including antitumor [3], antinociceptive [4], antihypertensive [5], and antimyocardial ischemia [6]. *levo*-Tetrahydropalmatine (*l*-THP) is the main active ingredient of YHS [7], and purified or synthetic *l*-THP is approved for use and available as Rotundine or Rotundin in China [8]. Recently, *l*-THP has been reported to show toxic effects such as depression of neurological, respiratory, and cardiac function in pediatric poisonings, as well as acute or chronic hepatitis after regular use in adults [9, 10].

The effects of YHS on drug-metabolizing enzymes such as cytochromes P450 (CYPs), which are important in controlling xenobiotic metabolism, have occasionally been reported. CYPs is a group of hemoproteins that are important for oxidative metabolism (phase I) of clinically used drugs and other xenobiotics [11]. Co-administration of drugs that are metabolized by CYPs as well as those that induce or inhibit CYPs can cause drug–drug interactions, and these interactions may lead to severe effects [12]. A few alkaloid constituents of YHS have been reported to induce or inhibit CYPs. For example, tetrahydropalmatine (THP) inhibited recombinant human CYP2D6 (IC_50_ = 3.04 μM ± 0.26 μM) and recombinant human CYP3A4 (IC_50_ = 41.5 μM ± 3.8 μM) activities [13]; THP enantiomers were metabolized mainly by CYP3A4/5 and CYP1A2 in human liver microsomes (HLMs), and *l-*THP significantly inhibited CYP2D6 activity [14]; protopine and allocryptopin induce CYP1A1 expression but have no influence on its enzyme activity in HepG2 cells [15].

However, information about whether total alkaloid extract (TAE) from YHS influences CYPs *in vivo*, especially after repeated administration, is limited. In the present study, a range of dosages of TAE from YHS (0, 6, 30, and 150 mg/kg) were orally administered to male Sprague–Dawley rats. The effects of TAE on the expression of five drug-processing genes including CYP1A2, CYP2C11, CYP2D1, CYP2E1, and CYP3A1 were investigated. Moreover, CYPs activities and hepatotoxicity were characterized.

## Methods

### Plant material

The dried and pulverized tubers of *yanhusuo* were purchased from Tianjin Tongrentang pharmacy (Tianjin, China) in October by M. Li (department of pharmacognosy, Tianjin University of TCM, China). The voucher specimen was deposited at the Academy of Traditional Chinese Medicine of Tianjin University of TCM (No. 2012).

### Chemicals and reagents

Phenacetin (PHE), paracetamol (PAR), tolbutamide (TOL), 4-hydroxytolbutamide (OHTOL), dextromethorphan (DEXM), dextrorphan (DEXP), midazolam (MDZ), 1-hydroxymidazolam (OHMDZ), chlorzoxazone (CHL), 6-hydroxychlorzoxazone (OHCHL), β-nicotinamide adenine dinucleotide phosphate (NADPH) were purchased from Sigma Chemical Co. (St. Louis, MO, USA); carbamazepine (internal standard), phenobarbital (PB) were obtained from the National Institute for the Control of Pharmaceutical and Biological Products (Beijing, China). Methanol and acetonitrile were high performance liquid chromatography-grate from Concord Corporation (Tianjin, China). Ultra-pure water was obtained from a Milli-Q Pluswater purification system (Millipore, Bedford, MA, USA). All other regents were of analytical grade.

### Animals

Male Sprague-Dawley rats (age, 5-7 weeks; weight, 210-230 g) were purchased from the Laboratory Animal Science and Technology of Tianjin Shanchuanhong Co., Ltd (Tianjin, China, Certificate No. SCXK-2013-0004). All rats were kept under a standard 12-h dark/light cycle with water and food provided *ad libitum*. All procedures involving animals were conducted in conformity with the Animal Research: Reporting In Vivo Experiments (ARRIVE) guidelines, and were approved by the Academy of Military Medical Science Institutional Animal Care and Use Committee (Certificate No. SCXK620076004).

### Sample preparation and analysis

**Figure 1:**
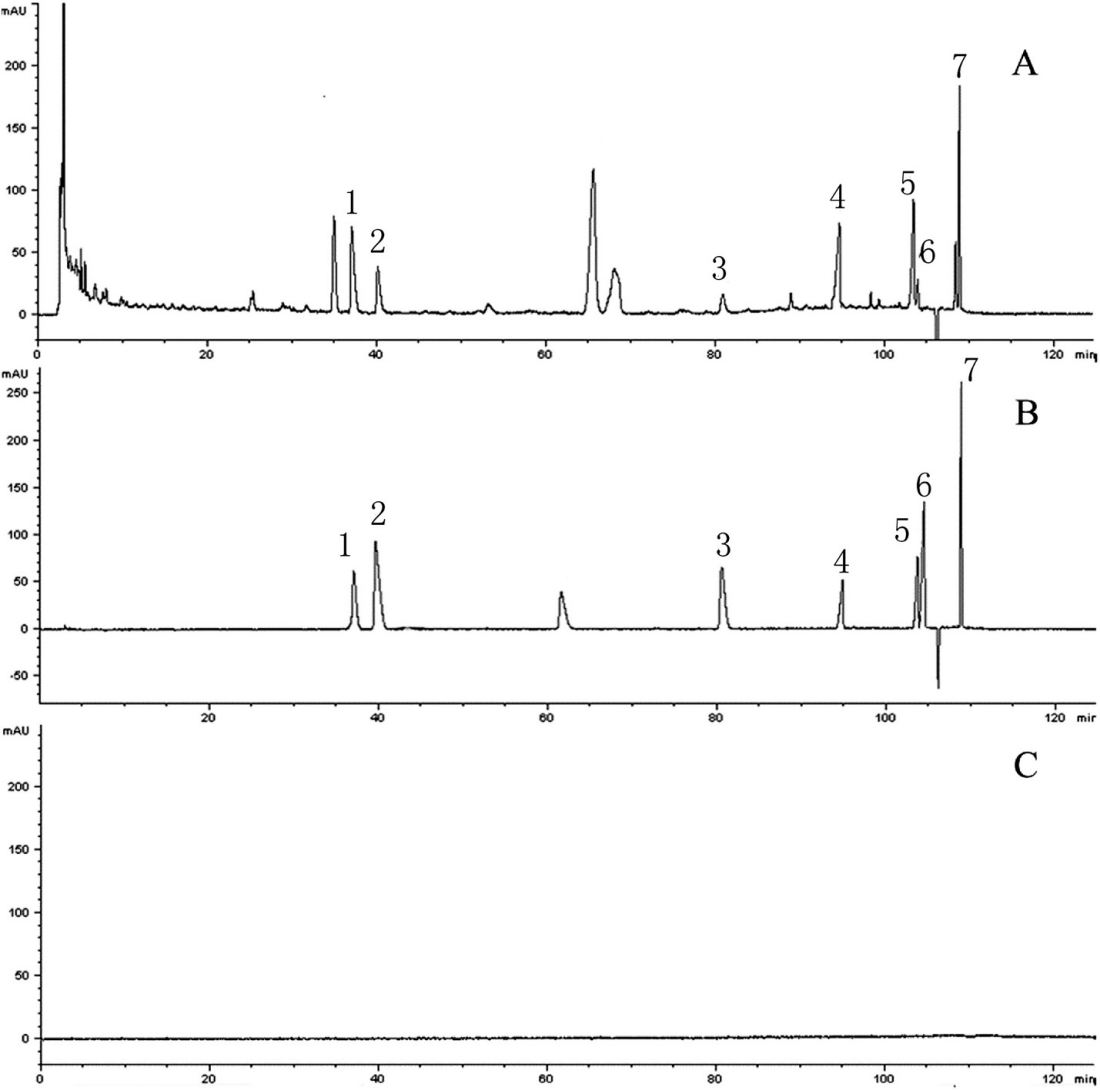
**HPLC-fingerprint chromatograms of total alkaloids of YHS. (A)** Chromatogram of the total alkaloid extract (TAE); **(B)** chromatogram of reference standards; and **(C)** chromatogram of the blank solvent; 1, protopine; 2, allocryptopin; 3, dehydrocorydaline; 4, tetrahydropalmatine; 5, corydaline; 6, tetrahydroberberine;7, glaucine.

**Figure 2:**
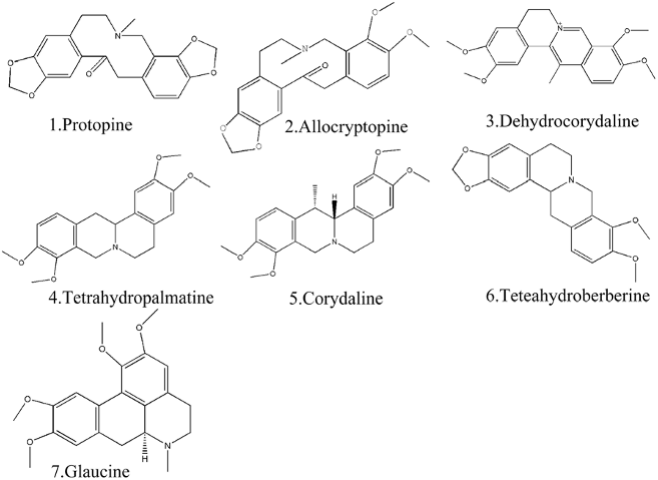
**Chemical Structure of the main alkaloid components of YHS.**

The dried and pulverized tubers of *yanhusuo* (2 Kg) were refluxed with 70% alcohol for three times. The pooled extract was concentrated under vacuum drying at 50°C. The dry powder was then dissolved in 200 mL water. The extracted solution was purified using 732 strong acid cation exchange resin, and the following elution program was used: six times water, and six times 70% ethanol:water containing 5% ammonia. A solution of 70% ethanol:water containing 5% ammonia was obtained, and the solvent was evaporated under reduced pressure. Preliminary analysis of the extracts was performed using an Agilent 1200 series high performance liquid chromatography (HPLC) instrument (Agilent Technologies, USA). Chromatographic separation was performed using an Agilent Zorbax HC-C_18_ (4.6 mm × 250 mm × 5 μm) column (Agilent, USA). The mobile phase were (A) 0.5% phosphate buffer (pH 5.0) and (B) methanol and the elution system was as follows: 0 - 25 min, from 85% A to 70% A; 25 - 75 min, from 70% A to 65% A; 75 -103 min, from 65% A to 25% A; 103 - 105 min, from 25% A to 20% A; 105 - 115 min, from 20% A to 85% A;115 - 125 min, 85% A. The flow rate was 1.0 mL/min, and the UV wavelength was 280 nm. The major alkaloids in YHS extract were quantified, and HPLC (Figure 1) showed that the contents (w/w) of protopine, allocryptopin, dehydrocorydaline, tetrahydropalmatine, corydaline, tetrahydroberberine, and glaucine were 2.52%, 1.67%, 0.34%, 3.58%, 3.0%, 0.22%, and 0.66%, respectively. Figure 2 presents the structures of the alkaloid components. The total alkaloid content (>45% in the extract) was determined by UV spectroscopy.

### Experimental procedure

Rats were randomly divided into 5 groups (total 50 rats, n = 10): three TAE-treated groups, the control group, and the positive control group, respectively. In 14 consecutive days, TAE-treated groups were given TAE orally at doses of 6, 30, 150 mg/kg once daily, the positive control group was intraperitoneal injection with phenobarbital (80 mg/kg) once daily [16], whereas the control group was orally with equivalent 0.5% sodium carboxymethylcellulose solution (5 mL/kg) once daily. After two weeks, the rats were quickly anaesthetized with urethane (140 mg/kg, 20% w/v solution in 0.9% NaCl) and then sacrificed by exsanguination of aorta abdominalis. The blood samples were centrifuged at 10000 g for 5 min at 4°C. Serum was collected immediately after each spin and stored at -80°C until the time of assay. Livers, kidneys, lungs and intestines were removed immediately and rinsed with physiological saline. Liver was cut into small pieces to get the liver tissue. Renal cortex was removed and minced in slices to get the kidneys tissue. Lung was cut into samll pieces to get the lung tissue. To get the intestinal mucosa, pieces of intestine were placed on an ice-cold glass plate, and the intestinal mucosa gently squeezed out. All the tissues were stored at -80°C for further analysis (the TAE dosage of 30 mg/kg was calculated to equivalent to the clinical dosage).

### Histopathology and blood biochemistry

Following the 14-day treatment, livers were removed from the rats, fixed in 10% neural buffered formalin for 48 h, processed, embedded in paraffin, sectioned at 4 μm, and stained with hematoxylin and eosin for histological evaluation. Incidence of hepatic lesions was determined by histologically examining the same liver section from each animal [12]. Serum alanine aminotransferase (ALT) levels were determined using a commercial kit, GPT-UV test Nanjing jiancheng (Nanjing jiancheng Bioengineering Institute). The potential hepatotoxicity to animals treated with different TAE dosages was determined.

### Microsomal CYPs activity detection

Microsomes were isolated from the rats livers (approximately 2 g) as described [17]. A rapid chromatography/tandem mass spectrometry (LC-MS/MS) method was applied for determination of the activity of five CYPs in the rat liver [18]. The HPLC system consisted of an LC-20 AD pump, a DGU-20 A3 degasser, an SIL-20 AC autosampler and a CTO-20A column oven (Shimadzu, Japan). The separation was performed on an Agilent ZORBAX XDB-C18 column (50 mm × 2.1 mm × 3.5 μm, Agilent). The flow rate was 0.45 mL/min and consisted of water with 0.1% formic acid (A) and methonal with 0.1% formic acid (B) using a gradient elution (0.0 - 0.5 min, 98% A; 0.5 - 1.0 min, from 98% A to 2% A; 1.0 - 2.5 min, 2% A; 2.5 - 2.51 min, from 2% A to 98% A; 2.51 - 4.0 min, 98% A) were used for analysis. An API 4000 Qtrap mass spectrometer (Applied Biosystems, Foster City, CA, USA) equipped with electrospray ion (ESI) source was used for mass analysis and detection. Following optimization of the setting parameters, the ESI source was operated in both poitive mode for (PAR, DEXP, OHMDZ, OHTOL, and carbamazepine) and negative mode for (OHCHL) with the curtain, nebulizer and turbo-gas (all nitrogen) set at 15, 60 and 55 psi, respectively. The source temperature was 550°C and the ion spray needle voltage was 5000 V in poitive mode and -4200 V in negative mode. PHE (CYP1A2), TOL (CYP2C11), DEXM (CYP2D1), CHL (CYP2E1), and MDZ (CYP3A1) were selected as the CYPs isoform probe substrates for the current study. The multiple reaction monitoring (MRM) mode was chosen for quantification of the metabolites of the probe substrates (Table 1). The recovery (extraction efficiency) of PAR, OHTOL, DEXP, OHCHL, and OHMDZ from HLMs were determined at three different concentrations of quality control samples ( they were 0.1, 0.5, and 2.5 μM). The internal standard was carbamazepine (75 ng/ml). The extraction recoveries of PAR, OHTOL, DEXP, OHCHL and OHMDZ were 86.3 ± 2.0%, 92.1 ± 1.6%, 87.2 ± 1.3%, 93.3 ± 2.0%, and 90.8 ± 1.9%, respectively.

**Table 1:** **MRM transitions and collosion energies for the detection of CYPs probe substrate metabolites**

Substrate metabolite	Molecular mass (MW)	Precursor (m/z)	Product (m/z)	Polarity	Collision energy (eV)
Paracetamol (PAR)	151	152	110	ESI^+^	23
Dextrorphan (DEXP)	257	258	199	ESI^+^	38
1-Hydroxymidazolam (OHMDZ)	341	342	203	ESI^+^	40
4-Hydroxytolbutamide (OHTOL)	286	287	171	ESI^+^	59
6-hydroxychlorzoxazone (OHCHL)	185	184	120	ESI^-^	-26
Kamaxiping (internal standard)	236	237.	194	ESI^+^	40

Incubation mixtures (200 μL) containing 0.1 M potassium phosphate buffer (pH 7.4), 0.25 mg/mL rat microsomal protein, and 10/100/2.5/20/5 μM PHE/TOL/DEXM/CHL/MDZ were preincubated for 5 min at 37°C in a water bath with gentle shaking. The reaction was initiated by addition of 1 mM NADPH and terminated with 400 μL chilled methanol (contained the internal standard of carbamazepine, 75 ng/ml) after 20 min incubation. After shaking (2 min) and centrifugation (5000 g, 10 min), the organic phase was separated and dried. The residues were dissolved in 200 μL of methanol and water (50:50, v/v) and centrifuged at 10000 g for 5 min. A 10 μL aliquot of supernatant was injected for determination of the metabolites of five CYPs probe substrates (PAR/OHTOL/DEXP/OHCHL/ OHMDZ) by LC-MS/MS.

### RT-PCR

Total RNA were extracted from the samples of liver (approximately 50 mg), kidney (approximately 50 mg), lung (approximately 50 mg) and intestine (approximately 50 mg) were extracted using Trizol reagent (Applied Biosystems, Foster City, CA, USA) according to the protocol provided by the manufacturer. The concentration of RNA was determined by spectrophotometry at 260 nm. RNA samples (2 μg) were reverse transcribed using the High Capacity cDNA Reverse Transcription Kit (Applied Biosystems, USA) to obtain cDNA according to the manufacture^,^s recommendations. Quantification of mRNA was performed on the Applied Biosystem 7500 Real-Time PCR System (Foster, CA, USA) using SYBR Select Master Mix (Applied Biosystems, USA) according to the manufacture^,^s recommendations. PCR reactions contains an initial denaturating cycle at 95°C for 10 min , followed by 40 amplification cycles: 95°C for 15 s and 60°C for 1 min. The primer pairs for five CYPs and β-actin genes and predicted size of PCR products are shown in Table 2.

**Table 2:** **Sequences of the primers in reverse transcription and quantitative real-time PCR**

Gene name	Sequence	Product size (bp)
β-actin		
Forward	ACCCCAAAGCCAACAGAGAG	102
Reverse	AGGCATACAGGGACAGCACA	
CYP1A2		
Forward	CATAGCCTCAGACCCCACAT	165
Reverse	ATGGCTCCGATGACATTAGC	
CYP2C11		
Forward	AGGGCCTTGGAGTCATTTTT	163
Reverse	GCACCTTTGCTCTTCCTCAG	
CYP2D1		
Forward	GCTGACAAGGTCTTCCAAGG	188
Reverse	ACCACCATGCGTAGGTTCTC	
CYYP2E1		
Forward	ATGTCATCCCCAAGGGTACA	184
Reverse	AGGCCTTCTCCAACACACAC	
CYP3A1		
Forward	TATGGGGAAAGCCATCTCTG	164
Reverse	CAGGTTTGCCTTTCTCTTGC	

### Statistical analysis

Values are expressed as mean ± SD. The enzyme activity was calculated as follows: enzyme activity = C_metabolite_ × L_incubation_/T_incubation_ /(C_HLMs_ × L_incubtiaon_), in which C_metabolites_ represents the concentration of metabolites of CYPs probe substrates, L_incubation_ represents the volume of incubation system, T_incubation_ represents the incubation time, and C_HLMs_ represents the concentration of HLMs. Values were expressed in units of pmol · min^-1^ · mg^-1^. Independent-sample t-test was used to analyze the differences between the enzyme activities, gene expression values with control values by SPSS 13.0. Differences were considered significant at *P* < 0.05.

## Results

### Liver function of TAE-treated rats

**Figure 3:**
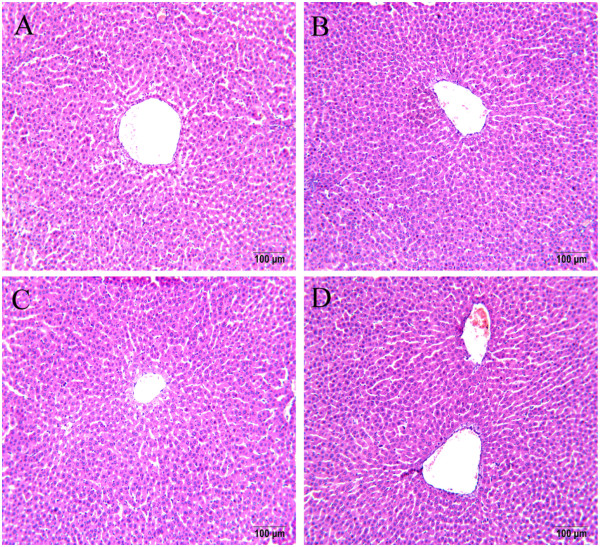
**Histopathological examination of liver sections from rats treated with TAE from YHS.** Liver sections of male Sprague–Dawley rats treated with various dosages of TAE showed largely normal appearances. For each dosage group, four sections were made, and the microphotos show representative foci in the control rats **(A)**, 6 mg/kg **(B)**, 30 mg/kg **(C)**, and 150 mg/kg **(D)** TAE-treated rats. Tissues were fixed in formalin and stained with hematoxylin and eosin. Magnification: 200 × .

No significant change was observed in serum ALT levels in the TAE-treated rats (data not shown). Figure 3 presents the histopathology microphotos. On histopathological examination, the control rats showed clear dividing lines of hepatic lobules. Their structure was complete, and the hepatic cord was orderly and was arranged radially around a central vein (Figure 3A). Liver portions from the TAE-treated rats (Figure 3B–D) did not show morphological alterations relative to the control rats (Figure 3A).

### Influence of TAE on CYPs activity in the rat liver

After TAE treatment, CYPs activities in the rat liver were quantified using a cocktail of probe drugs and LC-MS/MS. TAE significantly increased the enzyme activities of CYP2E1 and CYP3A1. In the treated rats, the three TAE dosages (6, 30, and 150 mg/kg, daily) significantly increased the enzyme activity of CYP2E1 by 20%, 68.2%, and 146.1%, respectively, and the three TAE dosages increased the levels of CYP3A1 by 27.84%, 40.3% and 51.9%, respectively, compared with those in the control rats. The highest TAE dosage (150 mg/kg, daily) increased the enzyme activity of CYP1A2 by 44.9% and the enzyme activity of CYP2C11 by 35.4%. The three TAE-treated groups were not significantly different from the control groups in terms of CYP2D1 expression (*P* > 0.05; Table 3).

**Table 3:** **Effects of TAE from YHS on CYPs activity in the rat liver**

	Dosage	The activities of CYPs (pmol · min ^-1^ · mg ^-1^)				
Group	(mg · kg ^-1^)	1A2	2C11	2D1	2E 1	3A1
Control		107.67 ± 2.36	116.00 ± 3.77	125.78 ± 9.05	299.33 ± 23.57	175.56 ± 3.35
Positive control		152.67 ± 2.82^**^	172.67 ± 2.83^*^	135.40 ± 1.50	930.00 ± 21.40^**^	351.00 ± 0.47**
TAE	6	97.78 ± 2.69	107.33 ± 0.67	120.67 ± 4.16	358.89 ± 23.65^*^	224.44 ± 1.92*
	30	107.56 ± 9.46	112.22 ± 6.68	121.56 ± 6.74	503.33 ± 21.86^**^	246.33 ± 2.36*
	150	155 ± 9.90^**^	157.11 ± 3.67^*^	125.78 ± 3.79	736.67 ± 14.14^**^	266.67 ± 5.66*

Data are presented as mean ± SD of three rats. *P < 0.05, **P < 0.01, values significantly different from the control rats.

### Regulation of CYP2E1 and CYP3A1 mRNA by TAE in the rat liver, kidney, lung, and intestine

**Figure 4:**
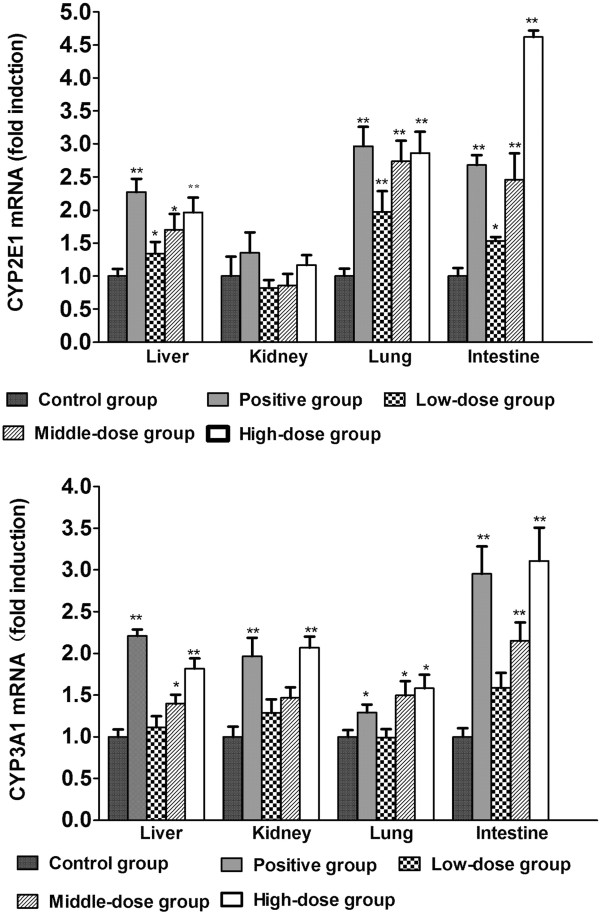
**Relative changes in the gene expression levels of CYP3A1 and CYP 2E1 by TAE from YHS in the rat liver, kidney, lung, and intestine.** Rats were treated for 14 days with TAE, phenobarbital (positive control), or 0.5% sodium carboxymethylcellulose solution (control). The mRNA levels of CYP3A1 and CYP2E1 were determined by real-time PCR with the results normalized to those of β-actin (as indicated). Data was expressed as mean ± SD triplicate measurements of five samples. *P < 0.05; **P < 0.01, response significantly increased relative to the control group.

Figure 4 presents the effects of TAE on the mRNA levels of CYP2E1 and CYP3A1. TAE at 6 and 30 mg/kg markedly increased the mRNA levels of CYP2E1 and CYP3A1, respectively, whereas TAE at 150 mg/kg increased the mRNA levels of CYP3A1 in the rat kidney. The mRNA levels of CYP2E1 in the rat kidney remained unchanged. At the highest dosage, the mRNA levels of CYP2E1 increased 2.0-, 2.9-, and 4.6-fold in the rat liver, lung, and intestine, respectively, when normalized to the mRNA level of β-actin. The gene expression levels of CYP2E1 and CYP3A1 increased dosage dependently.

### Regulation of CYP1A2, CYP2C11, and CYP2D1 mRNA by TAE in the rat liver, kidney, lung, and intestine

**Figure 5:**
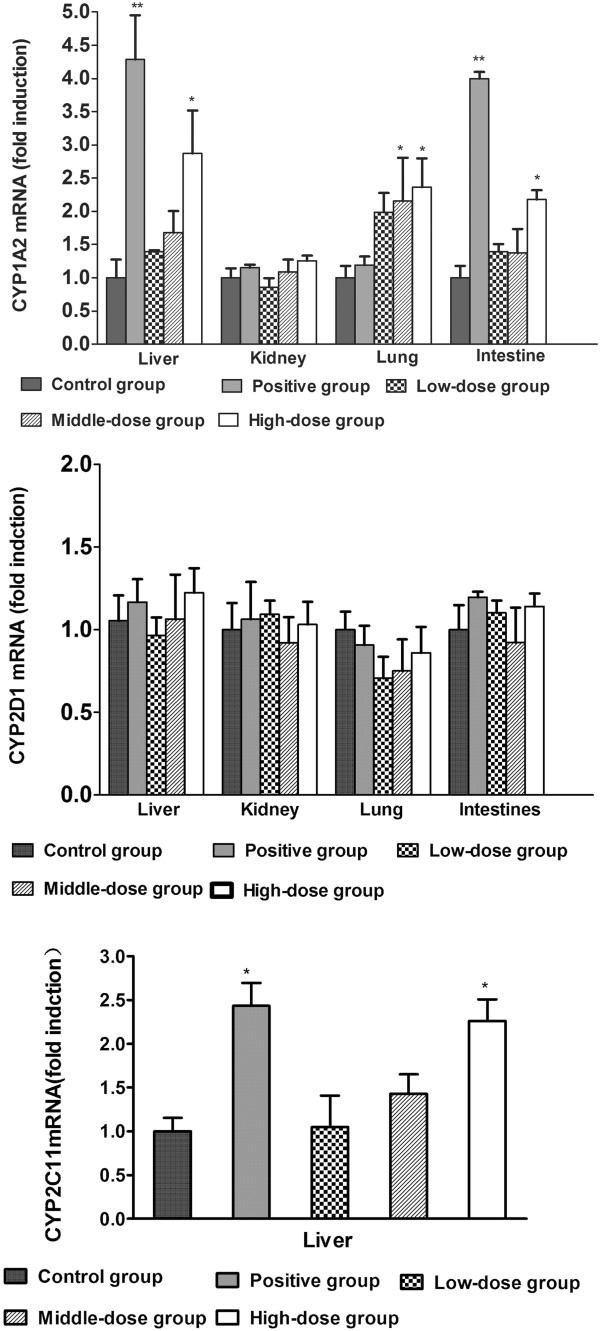
**Relative changes in the gene expression levels of CYP1A2, CYP2D1 and CYP2C11 by TAE from YHS in the rat liver, kidney, lung, and intestine.** Rats were treated for 14 days with TAE, phenobarbital (positive control), or 0.5% sodium carboxymethylcellulose solution (control). The mRNA levels of CYP1A2, CYP2D1, and CYP2C11 were determined by real-time PCR with the results normalized to those of β-actin (as indicated). Data was expressed as mean ± SD triplicate measurements of five samples. *P < 0.05; **P < 0.01, response significantly increased relative to the control group.

Changes in the mRNA levels of CYP1A2, CYP2C11, and CYP2D1 were evaluated by real-time PCR. As shown in Figure 5, TAE increased the mRNA levels of CYP1A2 at the highest dosage and showed 2.9-, 2.4-, and 2.2-fold increase in the CYP1A2/β-actin mRNA levels in the rat liver, lung, and intestine. No effect of the two lower dosages of TAE was observed on the gene expression levels of CYP2C11 (*P* > 0.05), and only up-regulation of the gene expression levels of CYP2C11 at the highest dosage was significant in the rat liver, which showed 2.3-fold increase in the CYP1A2/β-actin mRNA levels. We were unable to detect changes in the mRNA levels of CYP2C11 in the rat kidney, lung, and intestines because of the low gene expression levels of CYP2C11 in these organs. The mRNA levels of CYP2D1 did not change significantly among the three TAE dosages.

## Discussion

### CYP2E1 induction associated with TAE toxicity

In traditional Chinese herbal medicine, YHS is generally used to dispel stasis and move *qi*, reinforce vital energy, and relieve painful conditions such as headache, chest pain, and abdominal pain [19]. The main active ingredient of YHS is alkaloids; therefore, it is necessary to elucidate the possible interactions of alkaloids with the metabolism of drugs and other xenobiotics.

To date, some compounds in YHS have been found to exert the inhibitory or inductive effects on CYPs [20, 21], but little is known about the effect of TAE from YHS on CYPs expression. The present study was designed to investigate whether TAE from YHS affects the expression of CYP1A2, CYP2C11, CYP2D1, CYP2E1, and CYP3A1 in rats.

CYP2E1 catalyzes the biotransformation of almost 2% of all clinically used drugs in humans [22]. Rat CYP2E1 is a homolog of human CYP2E1 [23] and is expressed in many tissues including the liver [11]. CYP2E1 is a classical ethanol-inducible CYPs and has been extensively studied for long because of it catalyzes the bioactivation of several procarcinogens and protoxins including N-nitrosodimethylamine, benzene, and carbon tetrachloride [24]. CYP2E1-mediated metabolism generates reactive oxygen species, such as oxygen and hydroxyl radicals, when these exceed the cellular detoxification systems, it results in oxidative stress with its various pathologic consequences [11]. Oxygen radicals play a key role in liver injury because of their interaction with cellular proteins or DNA [11, 25]. CYP2E1 overexpression generated oxidative stress in a human hepatoma cell line and induced cytotoxicity to the cells [26], and CYP2E1 induction could alter immune system responses, leading to increased susceptibility to viral infection [27]. Furan is metabolized by CYP2E1 to a toxic metabolite, cis-2-butene-1,4-dial, that may interact with proteins to cause cytotoxicity or react with nucleosides to form substituted deoxyguanosine adducts [28].

Cases of human poisoning have occurred because of unregulated use of proprietary biopharmaceuticals containing purified THP. Several case reports associate the development of acute or chronic hepatitis with the chronic ingestion of a Chinese herbal medication “Jin Bu Huan Anodyne Tablets” [29, 30], which contains purified, concentrated *l*-THP, and the plant alkaloid responsible for its toxicity [9, 30]. In the present study, after the treatment of rats for 14 days with TAE, both the enzyme activity and mRNA level of CYP2E1 were significantly increased at all three TAE dosages. The liver injury caused by YHS may thus have resulted from the induction of the drug metabolic enzyme CYP2E1 by long-term administration of YHS. Drug-drug interactions are of concern when low-dosage TAE from YHS as well as substrates of CYP2E1 are administered.

### Induction ability of TAE on CYP3A1 in rats

The CYP3A subfamily is the most important hepatic metabolic enzyme in the metabolism of 40% to 60% of all drugs [31]. CYP3A4 is the most abundant CYP in the human liver, where it accounts for 30% of CYPs [22], and rat CYP3A1 is a homolog of human CYP3A4 [32]. CYP3A1 can catalyse the 6β-hydroxylation of testosterone [33] and the metabolism of a large variety of clinical medications, including many pediatric drugs [34], cyclosporin A [35]. Induction of CYP3A4 expression accelerated the clearance of several clinically important drugs including midazolam, amitriptyline, cyclosporin, and oral contraceptives [36]. Ginkgolide A and ginkgolide B induced the protein expression and enzyme activity of CYP3A in the primary cultures of human hepatocytes at 30 μmol/L [37]. In the present study, CYP3A1 was significantly induced in the rat liver, lung, and intestine at 30 mg/kg (equivalent to the clinical dosage), suggesting that TAE has the potential to produce CYP3A-mediated drug–drug interactions. Consumption of YHS or YHS-containing products with the substrates of CYP3A should be taken more attention because of the possibility of drug-drug interactions.

### The influence of TAE on other CYPs

After the treatment of rats for 14 days with different dosages of TAE, significant increases were observed in the mRNA expression and enzyme activities of CYP1A2 and CYP2C11 at 150 mg/kg TAE, but the mRNA levels and enzyme activities of CYP2D1 did not change significantly among the three TAE dosages. In the human liver, CYP1A2, CYP2C and CYP2D6 are involved in the metabolism of 4%, 16%, and 30%, respectively, of drugs on the market [11]. CYP1A2 is regulated primarily by the aromatic hydrocarbon receptor (AhR), and CYP1A2 is induced by AhR-mediated transactivation following ligand binding and nuclear translocation [38]. AhR activation and the significant induction of the enzyme activity of CYP1A can accelerate the biotransformation of different procarcinogens and promutagens to carcinogens and mutagens that bind covalently to important functional macromolecules such as DNA, resulting in the carcinogenic transformation of cells [12]. However, in the present study, only rats treated with high-dosage TAE showed an increase in the mRNA levels and enzyme activity of CYP1A2, indicating that TAE has low potential to produce CYP1A-mediated drug–drug interactions. Human CYP2C9 is the major CYP2C form, accounting for 60% of total human CYP2C [11]. Rat CYP2C11 is considered the counterpart of human CYP2C9 and metabolizes many drugs including S-warfarin and widely used nonsteroidal anti-inflammatory drugs such as diclofenac [39]. CYP2D1 is the rat orthologue of human CYP2D6, and CYP2D6 has been the most studied human genetic polymorphism in drug metabolism [11]. In vivo clearance of CYP2D6 substrates in poor metabolizers is generally much lower than in extensive metabolizers, leding to higher plasma concentrations and the potential for clinical toxicities with therapeutic doses [40]. Our results suggest that TAE can induce the mRNA levels and enzyme activity of CYP2C11 in the rat liver only at the higher concentrations tested, suggesting that clinically important CYP2C11-mediated drug-herb interactions are unlikely to be induced by TAE from YHS. And TAE did not affect CYP2D1 mRNA level and activity in three doses, the finding suggests that the use of products containing YHS may be considered safe when co-administration with CYP2D1 substrates.

## Conclusions

TAE from YHS significantly induced the mRNA expression and enzyme activity of CYP2E1 and CYP3A1 in the rat liver, lung, and intestine. Furthermore, enzyme activity correlated well with mRNA expression. The results of the present dose–response study in rats suggest that potential CYP2E1 and CYP3A drug-drug interactions are unlikely at clinical dosages of TAE, but need to be considered when high dosages of TAE or TAE-containing products are co-administered with substrates of CYP1A2 or CYP2C11. Complex herb (drug)-drug interactions may ensue from the co-administration of YHS with other drugs, which is mediated by CYP2E1 and CYP3A1 enzymes.

## Abbreviations

YHS: Yanhusuo
CYPs: Cytochromes P450
TAE: Total alkaloid extract
HLMs: Human liver microsomes
*l*-THP: *levo*- Tetrahydropalmatine
PHE: Phenacetin
PAR: Paracetamol
TOL: Tolbutamide
OHTOL: 4-hydroxytolbutamide
DEXM: Dextromethorphan
DEXP: Dextrorphan
CHL: Chlorzoxazone
OHCHL: 6-hydroxychlorzoxazone
MDZ: Midazolam
OHMDZ: 1-Hydroxymidazolam
NADPH: β-Nicotinamide adenine dinucleotide phosphate
PB: Phenobarbital
i.p: Intraperitoneal
AhR: Aromatic hydrocarbon receptor.
